# Citing a Data Repository: A Case Study of the Protein Data Bank

**DOI:** 10.1371/journal.pone.0136631

**Published:** 2015-08-28

**Authors:** Yi-Hung Huang, Peter W. Rose, Chun-Nan Hsu

**Affiliations:** 1 Department of Computer Science, National Taiwan University, Taipei 106, Taiwan; 2 Intel-NTU Connected Context Computing Center, National Taiwan University, Taipei 106, Taiwan; 3 RCSB Protein Data Bank, San Diego Supercomputer Center, UC San Diego, La Jolla, CA 92093, United States of America; 4 Division of Biomedical Informatics, Department of Medicine, UC San Diego, La Jolla, CA 92093, United States of America; Peking University Health Science Center, CHINA

## Abstract

The Protein Data Bank (PDB) is the worldwide repository of 3D structures of proteins, nucleic acids and complex assemblies. The PDB’s large corpus of data (> 100,000 structures) and related citations provide a well-organized and extensive test set for developing and understanding data citation and access metrics. In this paper, we present a systematic investigation of how authors cite PDB as a data repository. We describe a novel metric based on information cascade constructed by exploring the citation network to measure influence between competing works and apply that to analyze different data citation practices to PDB. Based on this new metric, we found that the original publication of RCSB PDB in the year 2000 continues to attract most citations though many follow-up updates were published. None of these follow-up publications by members of the wwPDB organization can compete with the original publication in terms of citations and influence. Meanwhile, authors increasingly choose to use URLs of PDB in the text instead of citing PDB papers, leading to disruption of the growth of the literature citations. A comparison of data usage statistics and paper citations shows that PDB Web access is highly correlated with URL mentions in the text. The results reveal the trend of how authors cite a biomedical data repository and may provide useful insight of how to measure the impact of a data repository.

## Introduction

Consistent practice of data citation facilitates and incentivizes data sharing and reuse because it could be counted as professional recognition for data providers as citations of journal and other types publications. However, currently no commonly agreed data citation practice has been adopted. It is not clear which practice standard or policy gains the most adoption, nor is how they reflect the impact of the data being cited. The Protein Data Bank (PDB) [1–8] is the worldwide repository of experimentally determined structures of proteins, nucleic acids, and complex assemblies, including drug-target complexes. The PDB annotates structures according to standards set by the wwPDB [9] and provides unique identifiers and DOIs for its datasets. All journals require a prior submission of structures to the PDB as part of the publication process. This well matured process can serve as a model of data citation for other data initiatives. The PDB’s large corpus of data (> 100,000 3D structures) and related citations provides an extensive test set for developing data citation and access metrics. An important aspect is the interplay of literature and data citations, and the relative importance of these two mechanisms to make data discoverable. The analysis of the literature and data citation cascades demonstrates potential discovery pathways, that is, how knowledge and data were used to advance a particular field of science (*e.g.*, the discovery of HIV drugs).

This idea is carried out as a pilot project in bioCADDIE, an NIH BD2K (Big Data to Knowledge initiative) Data Discovery Index Coordination Consortium (https://biocaddie.org). The major aim of the pilot project is to analyze the characteristics of the paper and data citation networks of PDB to recommend data citation and provenance practices, approaches to discover data citations, methods of linking citations and data, and data access metrics, for the NIH Data Discovery Index. In this paper, we focus on analyzing citations to the PDB data repository. We will then investigate citations to individual structures as our next step. PDB users currently have different choices to cite the PDB data repository. They can cite the original debut publication of the RCSB PDB published in 2000 [1] (hereinafter, “the PDB debut paper”), which was highly cited, ranked 92 among the top 100 most-cited research of all time [10] with 12,754 citations. Alternatively, PDB users can cite one of the follow-up update papers of PDB published in the annual Database Special Issue of Nucleic Acids Research (NAR) from year 2002 to 2008 [2–8] and in other venues [9, 11–14]. These publications describe the progress of continued enhancement and development of PDB. Citing journal publications represents a traditional method of data citation, with the benefit of being persistent and unambiguous. Alternatively, PDB users can cite PDB by mentioning URLs linking to the PDB home pages on the Web in the text, like “(http://www.rcsb.org).” URLs are unique but not persistent. Also, URL mentions are hardly recognized as academic accomplishment. In addition to URL mentions, data usage statistics, such as download counts, is proposed to be considered to measure the impact of research works [15]. This paper aims to answer the following questions:

- Does a new PDB publication by any of the wwPDB members attract more new citations and does a new PDB publication decrease the growth of citations and influence of its predecessors?
- Do PDB users refer to PDB URLs more often than citing PDB publications? How many use both? If we consider URLs and PDB publications as independent works, do URLs decrease the growth of citations and influence of PDB publications?
- How does data usage statistics correlate to paper citations and URL mentions?

Our main analysis tool is the citation cascade analysis. Citation cascades are chains of citations between two articles in a citation network. Citation cascades can be quantified by a function that considers both the length of the chain and the number of paths. Previously, we have shown that the growth of citation cascades correlate with the lasting influence of research articles better than citation counts [16], which usually favor an old paper because it takes long to accumulate citations for a new paper to be considered more influential than an old one. In contrast, disruption of citation cascades of an established paradigm can serve as an early indicator of paradigm shift [17].

Data citation is receiving increasing attention in all disciplines of science as data become essential and ubiquitous in research. CODATA/ITSCI Task Force on Data Citation published a report on the current state of data citation in 2013 [18]. FORCE 11 (http://www.force11.org) has its final release of *Joint Declaration of Data Citation Principles* in 2014 [19], which identifies six principles as the guideline for the design of data citation standards and practices.

A few studies have focused on automatically connecting the citation patterns that are resident in the literature data to the biomedical databases. BioLit [20] provided a comprehensive view on the literature data that links to biomedical databases by integrating the content of PubMed Central (PMC) with that of the PDB repository, based on the text-mining approach. Şenay [21] characterized the patterns of how PDB entries are cited in research articles, based on analysis of the full text literature data available from Europe PubMed Central. Aur*é*lie [22] developed a framework that improves links between literature data and various biomedical databases.

Much of bibliometric analysis uses traditional academic citations to measure a paper’s quality or scientist’s productivity [23]. Beyond simple citations counts, researchers have explored methods that analyze the structure of citation networks to identify important papers [16, 24] or predict which papers will be important in the future [25]. Moreover, Lovro implemented a network-based statistical comparison of the citation topology for analyzing the consistency of various bibliographic databases [26]. Our analysis method differs from related work in that we consider cascades, which take chains of citations, into account. It is well known that citation counts decay over time even for a highly influential work [27]. Therefore, it is important to consider its continuing influence of cascades, which provide indirect exposure to the work. Ghosh and Lerman [28] developed a function to quantify the structure of a growing cascade of information spreading in social media, which we use to measure the size of evolving cascades. We have developed a preliminary approach to quantifying transformative research with a disruption score that based on this model.

One of the technical challenges is how to quantify and compare the influence of the PDB publications and URL mentions. Our approach to quantifying influence allows us to overcome this challenge by constructing citation cascades originated from papers with URL mentions. In this way, influence of URL mentions and PDB publications can be normalized and comparable, though cares must be taken in matching PMC full-text data, where URL mentions can be observed, with the PubMed citation network data set, where only abstracts are available.

## Materials and Methods

### Paper Citation Data

The citation data used in our study were collected from MEDLINE \ PubMed database (http://www.ncbi.nlm.nih.gov/pubmed) through the Entrez system and the XML format files from the NLM’s FTP sever (http://www.nlm.nih.gov/bsd/licensee/access/medline_pubmed.html). Each record contains XML elements <CommentsCorrections>. The attribute RefType=“Cites” of the element lists references or the bibliography of an article, from which we can obtain the citation information (see http://www.nlm.nih.gov/bsd/licensee/elements_descriptions.html). Our data set contains totally 21,483,488 articles and 93,860,986 pairs of cited-citing relation from PubMed, obtained in May 2014.

### Mining URL Mentions

We extracted and counted articles containing mentions of PDB URLs from the full-text article data available from PubMed Central (PMC). The data is available for download from (http://www.ncbi.nlm.nih.gov/pmc/tools/ftp/), in either NXML markup language or plain text. We obtained 782,890 articles in NXML format as of October 2014, and 967,022 articles in plain text format as of February 2015. Removing duplicate PMC IDs yielded a total of 972,725 articles.

We extracted mentions of URLs linking to the home pages of the wwPDB partners, including RCSB PDB, PDBe (PDB Europe) and PDBj (PDB Japan), and wwPDB (world-wide PDB). Table 1 shows the patterns that we used to extract URL mentions from the text. URLs that link directly to a landing page of a protein structure in PDB are excluded. These can be recognized by certain suffix patterns in the URLs, as given in Table 1. Formal URL citations, that is, citing PDBs as a paper citation and listing a URL in the bibliography section, were not considered. URLs that are DOIs (digital object identifiers) (http://www.doi.org) [29] were not included here, either.

**Table 1:** Text patterns considered as PDB URLs.

PDB site	URL	Inclusion Prefix	Exclusion Suffix
RCSB PDB	http://rcsb.org, http://www.rcsb.org, http://www.pdb.org	“*rcsb.org”, “*pdb.org”	“structureId=*”
wwwPDB	http://www.wwpdb.org	“*www.wwpdb.org”	
PDBe	http://pdbe.org, http://www.ebi.ac.uk/pdbe http://www.ebi.ac.uk/msd	“*pdbe.org”, “*www.ebi.ac.uk/pdbe”, “*www.ebi.ac.uk/msd”	“/entry*”
PDBj	http://pdbj.org	“*pdbj.org”	“/mine*”

### Citation Cascade and Disruption

In this study, we consider the RCSB PDB debut paper as the seed and use the *calibrated disruption score* to quantify how much of its influence is disrupted by PDB follow-up update papers, papers describing competing data repositories, and papers that cite PDB by URL mentions. In general, given a pair of papers, we can compute their calibrated disruption score from the citation network connecting to the pair of papers to quantify the disruption of the influence of the seed paper by a challenger paper. The computation can be applied when the seed and challenger are two collections of papers when we want to quantify how one group of the papers as the challenger collectively disrupt the influence of the seed. That is, either the seed or the challenger may consist of multiple papers.

A citation network is essentially a directed graph *G* = (*V*, *E*), where *V* is the set of papers and *E* is the set of edges indicating citations made by papers. A link (*i* ← *j*) ∈ *E* denotes that paper *j* cites paper *i*, *cite*(*j*) denotes the set of all papers that *j* cites and *cited*(*i*) the set of all papers that cite *i*. *V*_*t*_ is the set of papers published at time *t*.

Given one or more papers *S* ∈ *G*, a cascade *C* is a subgraph that contains all citation chains that end at *S*. The set *S* is called the *seed* or *root* of the cascade. The seed indirectly exerts influence on all papers in the cascade, but influence decays with the distance to the seed. For a node *j* in the cascade, the cascade generating function [28]*ϕ*(*j*) summarizes the structure of the cascade, *i.e.*, all citation chains, up to that point. The cascade generating function quantifies the influence of *S* on node *j*, and is defined recursively by

$$\begin{array}{c} \phi \left(j\right):=\{\begin{array}{cc} 1 & \text{if}\;j\in S\\ \sum_{i\in cite(j)}\alpha \phi \left(i\right) & \text{otherwise}, \end{array} \end{array}$$ (1)

where *α* is a constant damping factor. For a paper *j* published after *T* time steps (*e.g.*, years) from the publication of the seed, *ϕ*(*j*) can be written as $\phi (j)=\sum_{p=0}^{T}a_{p}\cdot \alpha^{p},$ where the coefficient *a*_*p*_ is the number of distinct paths of length *p* from one of the seeds to *j*. The impact of *α* is that the smaller the value of *α*, the higher the penalty against long paths. It is also possible to assign a unique *α*_*ij*_ for each link. Here we assigned a constant 0.8 for all links to control its impact.

**Fig 1:**
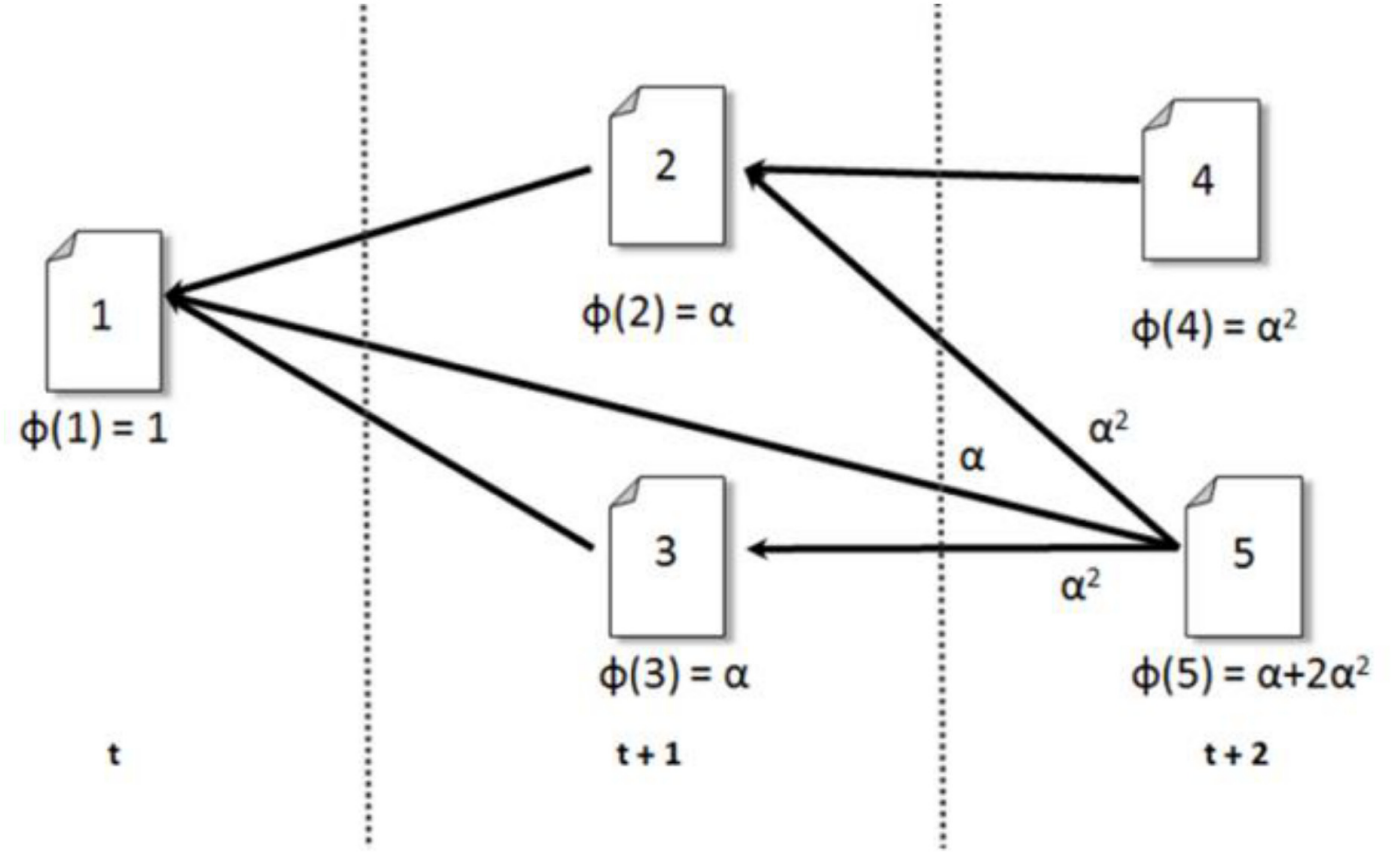
An example of cascade and *ϕ* values in a citation network.

Fig 1 shows an example cascade and the *ϕ* values for its nodes, where node 1 advocating some dominant scientific paradigm, and a link from node 2 to 1, means paper 2 cites paper 1. The cascade function *ϕ*(*j*) of a paper *j* is the sum of *αϕ*(*i*) for all papers *i* that *j* cites. For example, *ϕ*(5) = *αϕ*(1) + *αϕ*(2) + *αϕ*(3) = *α* + *α* ^2^ + *α* ^2^ = *α* + 2*α* ^2^.

We assume that if (*i* ← *j*) ∈ *E* and *i* ∈ *V*_*t*_ and *j* ∈ *V*_*t*′_ then *t* < *t*′. That is, no new paper should be cited by an older paper. Moreover, citations between papers published in the same time interval are not considered here. In this study, we chose the time interval *t* as a year. Therefore, citations between papers published in the same year are not considered. Such a citation network is a directed acyclic graph and cycles are deemed to be errors. From Eq (1), traversing the citation network in a topological order [30] and updating *ϕ* values along the way will guarantee that no backtracking is necessary to compute all *ϕ* values for all nodes. Therefore, we can apply topological sorting to compute *ϕ*. The time complexity of topological sorting is *O*(∣*V*_*C*_∣+∣*E*_*C*_∣), which is linear to the sum of the number of nodes and edges in a cascade *C*.

For example, the citation cascade of the RCSB PDB debut paper [1] contains 7,167,636 citations from our PubMed data set. These citations do not include 493,189 same-year citations to the papers in the cascade (6.4% reduction) to avoid cycles as explained above.

**Fig 2:**
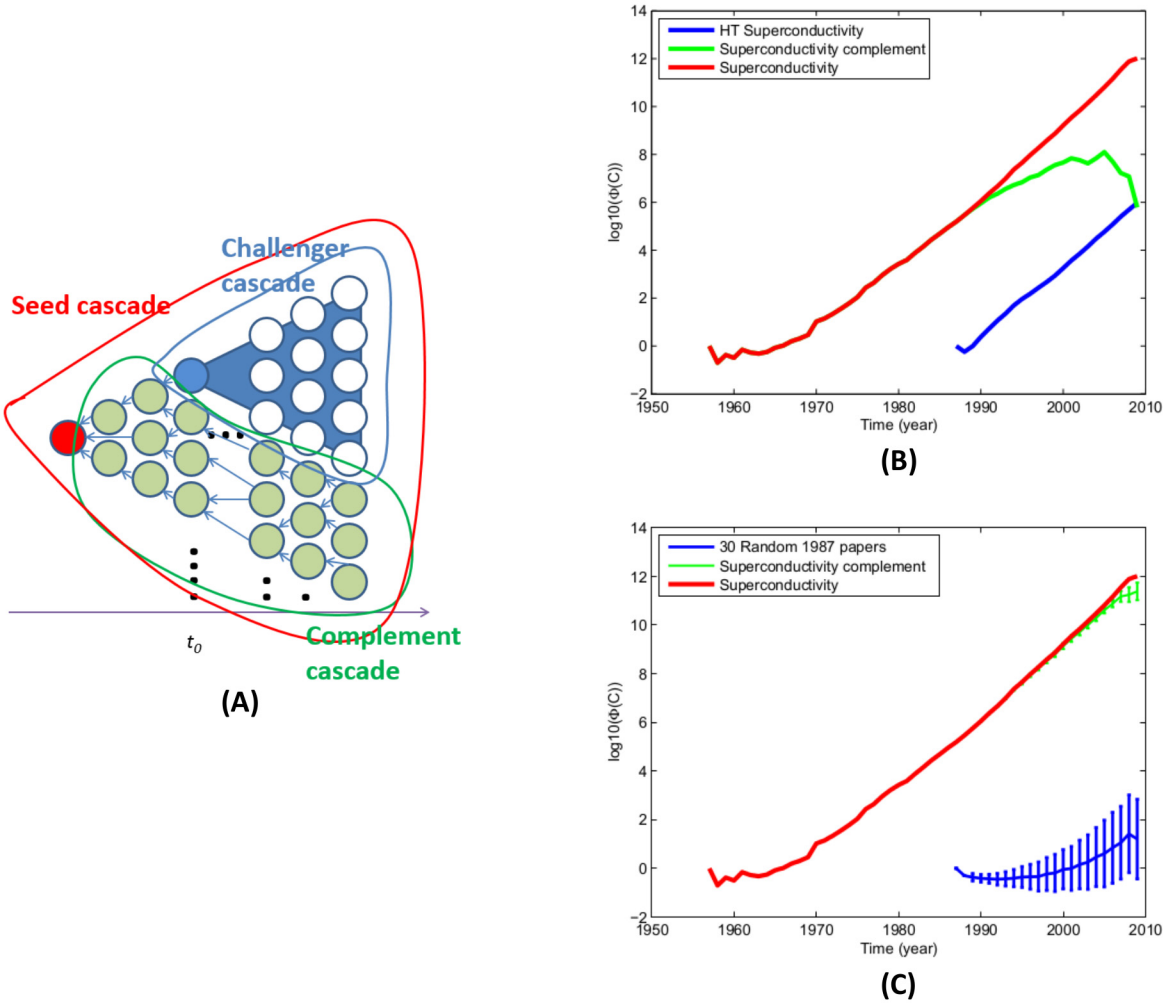
Disruption of the influence of the seed by a challenger can be observed in the changes to the citation cascade of the seed. (A) A citation cascade of a seed paper and residue cascade created by subtracting a challenger’s cascade. (B) An example of the disruption of the superconductivity theory (BCS) by the high temperature superconductivity theory (HTS). (C) 30 randomly selected papers published in the same year show no sign of cascade disruption to BCS, though the annual standard variation bars show that the growth of their own cascades are highly variant (blue curve). Meanwhile, the annual standard deviation bars of the growth of the residue cascades that they yield are small.

Changes of the *ϕ* value can be used to characterize the trend of influence changes of the seed [28] and quantify competing influence when two cascades overlap [17]. Fig 2(A) illustrates the idea. The cascade *C* of the seed paper (red node) is the network connecting all papers to the seed via citation. The seed that originates an established paradigm, marked in red, creates a cascade as it is cited by other papers, while a challenger, marked in blue, disrupts the growth of the cascade of the seed. The residue cascade is the complement of the seed cascade with nodes in the challenger cascade subtracted. When a challenger (blue node) is a paper that advocates a new paradigm, it attracts citations from papers in the cascade, shown as white nodes with blue background, leaving the residue cascade consisting of green nodes.

The disruption can be measured by comparing the growth of the average *ϕ* over time for all papers in the cascade and the papers in the complement of the cascade (green nodes). *C* is the entire cascade rooted by the seed paper. Let *C* ^(*c*)^ denote the cascade originating from the challenger. We define the *residue cascade*, denoted by $\tilde{C}$, as the complement subgraph of *C* obtained by subtracting *C* ^(*c*)^ from *C*, *i.e.*,

$$\begin{array}{c} \tilde{C}:=C-\left(C\cap C^{\left(c\right)}\right)=C\setminus C^{\left(c\right)}. \end{array}$$ (2)

By definition, references of papers in $\tilde{C}$ can only be traced back to the seed papers but not the challenger. We note that it is not necessary for the challenger to be in *C*. The blue nodes in Fig 2(A) are the root node(s) of the intersection of *C* and *C* ^(*c*)^.

Let *C*_*t*_ be the set of papers in cascade *C* published at time *t*. The average of the cascade function *ϕ* of papers in *C*_*t*_ is defined by

$$\begin{array}{c} \Phi_{t}\left(C\right):=\frac{1}{|C_{t}|}\sum_{j\in C_{t}}\phi \left(j\right)=\sum_{p=0}^{t}\bar{a_{p}}\cdot \alpha^{p}, \end{array}$$ (3)

where $\bar{a_{p}}$ is the average of the coefficient *a*_*p*_ in Eq (3) for *j* in *C*_*t*_, and $\bar{a_{p}}$ indicates on average number of distinct citation chains of length *p* from papers published at time *t* to the seeds. The variable Φ_*t*_ can be interpreted as an indicator of the seed paper’s influence at time *t*.

Consider an example of disruptive research of superconductivity in Physics that we studied previously [17]. The 1957 theory of superconductivity by Bardeen, Cooper, and Schriffer (BCS) [31, 32] was a dominant paradigm in this field until the discovery of high-temperature superconductivity [33] (HTS) in 1986, a disruptive research accomplishment for which the authors were awarded the Nobel Prize in Physics the next year. Fig 2(B) plots the growth of the logarithm of the annual average cascade function values Φ, defined in Eq (3), of the cascade of BCS. Without considering the challenger, it may appear that BCS continues to prosper, as its cascade continues to grow (red curve), but subtracting the part of the cascade taken over by HTS will reveal that the growth of the residue cascade (green nodes) slows and drops. In this case, the community’s attention shifts to new papers that support the challenger HTS.

To test the specificity of cascade interruption, we randomly selected 30 papers published in 1987, the same year as HTS seeds, from the APS dataset as negative controls and plotted the growth of their influence as shown in Fig 2(C), where the blue curve shows the means and standard deviations of the average cascades of these 30 challengers and the green curve shows those for their residue cascades. The curves show that though the growth of their cascades varies widely, the complements of the BCS cascade are hardly disrupted, unlike the HTS papers [17]. In general, we can visualize a disruption by a challenger if the curve that plots the growth of the residue cascade opens a gap between the curve of the growth of the seed cascade and starts dropping.

We can quantify the disruption in addition to visualization with a plot. Let *t*_0_ be the publication time of the challenger paper, the *calibrated disruption score* is defined as

$$\begin{array}{c} c\delta \left(\tau \right):=1-\frac{1}{\tau }\sum_{t=t_{0}+1}^{t_{0}+\tau }\frac{\sum_{j\in {\tilde{C}}_{t}}\phi \left(j\right)}{\sum_{j\in C_{t}}\phi \left(j\right)}. \end{array}$$ (4)

The calibrated disruption score is a revision of the disruption score [17] to normalize the range between 0 and 1 and ensure that scores of challengers published in different years are comparable when *τ* is set to the same value. Intuitively, a 5-year (*τ* = 5) calibrated disruption score greater than 0.7 amounts to a large portion of the new influence of the seed paper is indeed due to the challenger, suggesting that its influence has been disrupted by the challenger.

### Correlating Citations and Mentions with the PDB data usage statistics

The wwPDB provides monthly statistics of *FTP*, *Archive and Website Downloads*, and *Views* for each PDB structure from 2007 to present, available at (http://www.wwpdb.org/stats/download.php).

We searched for correlation between annual PDB usages and with the growth of the citation counts of the RCSB PDB debut paper and URL mentions. We used the least squares fit of a linear function to see if any linear relation that correlates a dependent variable *y* with an independent variable *x*:

$$\begin{array}{c} y\approx f(x)=w\cdot x+\beta . \end{array}$$ (5)

For example, let *u*(*t*) be a PDB usage statistics values in year *t* and *c*(*t*) be the citation counts or URL mentions in year *t*. We tested if they are correlated by assigning *y* = *u*(*t*) and *x* = *c*(*t*) and fit the linear function and vice versa. Furthermore, we also tested if any of them is a leading or lagging indicator of another count. For example, we fit a linear model with *y* = *c*(*t*) and *x* = *u*(*t* − 1) to test if usage statistics in a past year correlates with the citation count of a given year. We also consider different 2-year time frames by assigning, for example, *x* = *u*(*t*) + *u*(*t* − 1), the sum of the usage statistics in the current year *t* and the previous year *t* − 1, *etc.*

## Results

### Paper Citations

**Fig 3:**
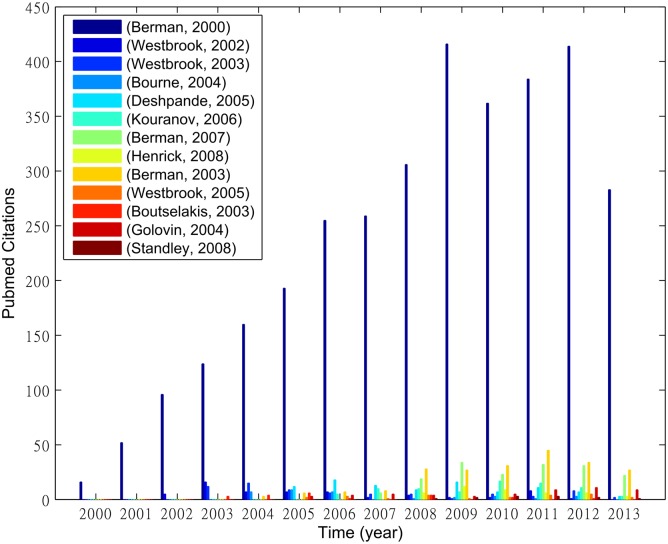
Citation growth of the debut PDB article and its follow-up articles.

We start by investigating whether authors choose to cite new PDB follow-up update papers instead of the RCSB PDB debut paper. We consider only those published before 2008 so that for every paper we can observe the growth of its citation counts for at least five years (up to 2013). Fig 3 shows that the annual citation counts of these PDB publications are much less than that of the highly cited PDB debut paper. The paper citation result seems to match the well-documented *Matthew effect* in science, which states that *the rich get richer and the poor get poorer* in terms of citations [34, 35].

**Fig 4:**
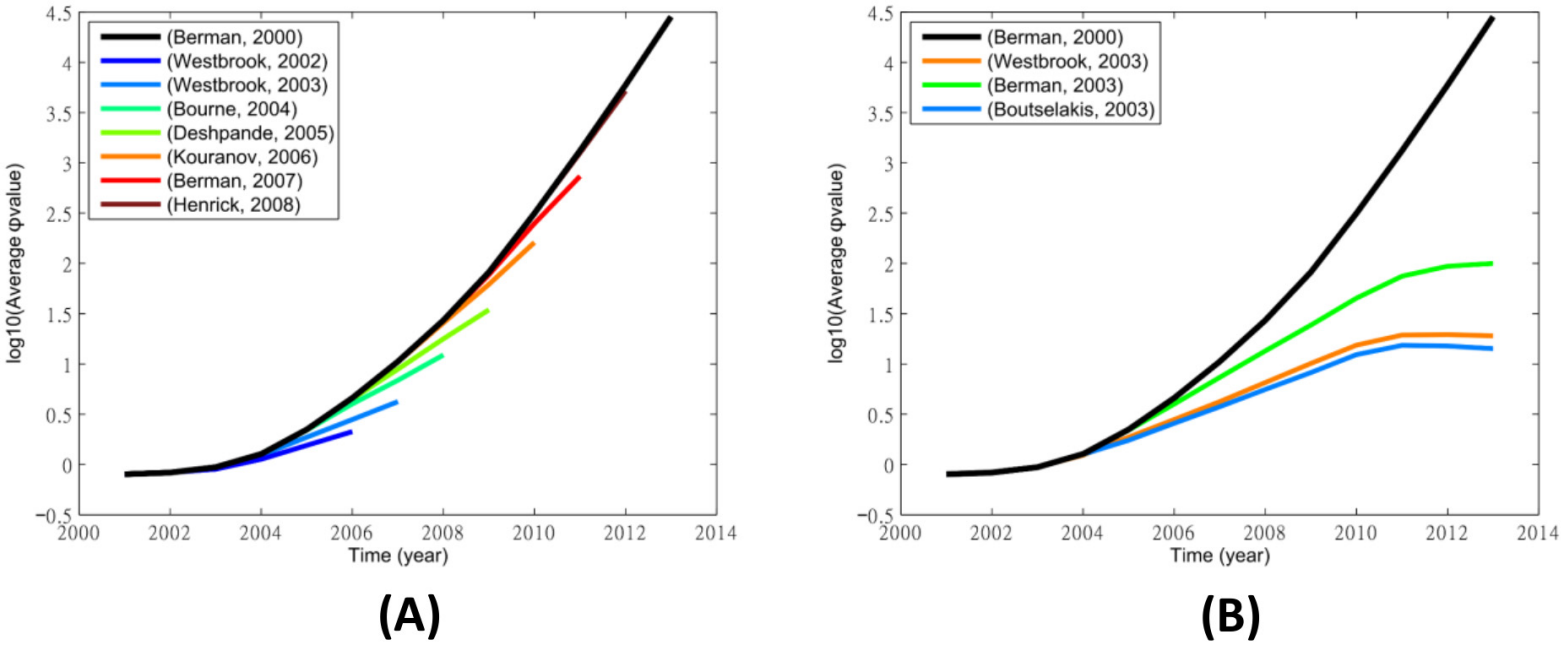
Compare the growth of the PDB debut paper’s cascade with (A) all the residue cascades created by its follow-up articles in 5 years (*τ* = 5) and (B) the residue cascades created by three 2003 follow-up articles. The y-axis of both panels shows the logarithm of the annual average cascade function values Φ, defined in Eq (3).

Though the citation counts of the follow-up update papers are not as large as the original debut paper, they may still disrupt the growth of the citation cascade of the PDB debut paper if they were cited by highly influential papers. To visualize if this is the case, we plot two graphs similar to Fig 2(B) to show the growth of the influence of the PDB debut paper and the growth of the residue cascades by the seven follow-up articles published in the Database Special Issue of NAR. Fig 4(A) shows that the growth of the residue cascade curves are close to the curve of the PDB debut paper after 5 years (*τ* = 5), suggesting that the follow-up articles hardly disrupt the growth of the cascade and thus the influence of the original PDB debut paper. Fig 4(B) compares long-term disruptions of three follow-up articles published in the same year (2003). The figure shows that the growth of these residue cascades start to open large gaps from the black curve but these curves of the residue cascades fail to drop downward, suggesting limited disruption to the influence of the original debut paper. Table 2 shows the calibrated disruption scores of all PDB follow-up articles published between 2002 to 2008. The first seven articles are those published in the Database Special Issue of NAR.

**Table 2:** 5-year calibrated disruption scores of the PDB follow-up articles. The last column shows the average scores of randomly selected papers published in the same issue.

Author	Year	Title	Calibrated Disruption Score	Avg of Random 5
Westbrook	2002	The protein data bank: unifying the archive [2]	0.34	0.01
Westbrook	2003	The protein data bank and structural genomics [3]	0.35	0.00
Bourne	2004	The distribution and query systems of the RCSB Protein Data… [4]	0.32	0.00
Deshpande	2005	The RCSB Protein Data Bank: a redesigned query system and … [5]	0.33	0.00
Kouranov	2006	The RCSB PDB information portal for structural genomics [6]	0.27	0.00
Berman	2007	The worldwide Protein Data Bank (wwPDB): ensuring a single… [7]	0.26	0.00
Henrick	2008	Remediation of the protein data bank archive [8]	0.10	0.00
Berman	2003	Announcing the worldwide Protein Data Bank. [9]	0.17	0.00
Boutselakis	2003	E-MSD: the European Bioinformatics Institute… [11]	**0.39**	0.00
Golovin	2004	E-MSD: an integrated data resource for… [12]	0.06	0.00
Westbrook	2005	PDBML: the representation of archival macromolecular… [13]	0.08	0.00
Standley	2008	Protein structure databases with new web… [14]	0.00	0.00

The last column of Table 2 shows the average scores of five randomly selected articles published in the same issue. The scores show that the follow-up articles still impact on the influence of the original debut papers much higher than other less related articles. We further compute the scores of the most highly cited articles in the Database Special Issues of NAR [36–42] in each year and show the results in Table 3. Again, none of them score very high but three articles related to protein and thus PDB [36–38] score higher than 0.4, which is higher than the scores of any follow-up papers of PDB, suggesting that these articles impose influence disruption to the PDB debut paper more than the PDB follow-up papers.

**Table 3:** 5-year calibrated disruption scores of the most highly cited articles in the database special issue of NAR.

Year	Title	Calibrated Disruption Score
2002	Gene Expression Omnibus: NCBI gene expression and hybridization … [39]	0.28
2003	The SWISS-PROT protein knowledgebase and its supplement TrEMBL … [36]	**0.53**
2004	The Pfam protein families database. [37]	**0.50**
2005	The Universal Protein Resource (UniProt). [38]	**0.42**
2006	miRBase: microRNA sequences, targets and gene nomenclature. [40]	0.29
2007	NCBI reference sequences (RefSeq): a curated non-redundant sequence… [41]	0.39
2008	The Pfam protein families database. [42]	0.30

### URL Mentions

**Fig 5:**
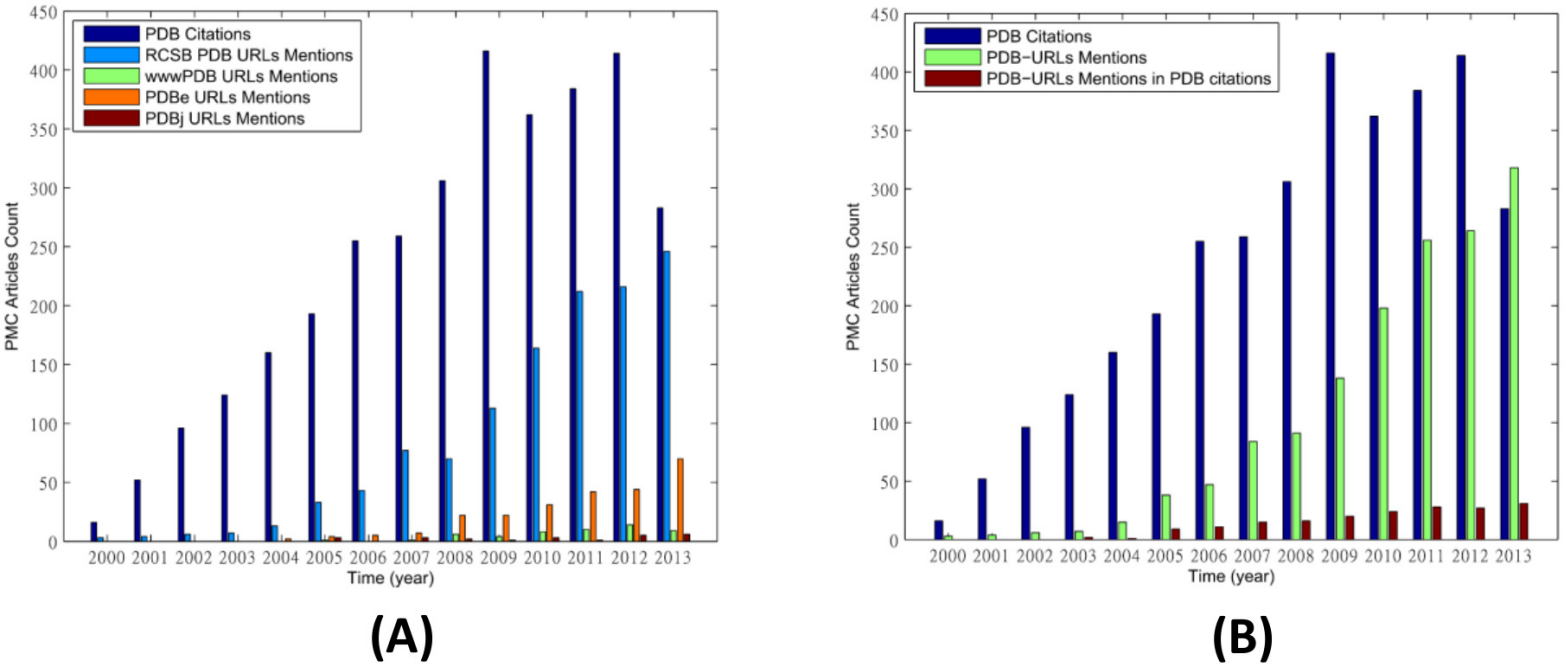
(A) Annual growth of the citations to the PDB debut paper and the counts of the different PDB URL mentions. (B) Annual growth of the citations to the PDB debut paper (blue bar), sum of all PDB URL mentions (green bar) and the count of the articles that not only directly cite the PDB debut paper but also mention PDB URLs (red bar).

We investigate the trend that authors mention PDB URL(s) in the text as data citation practice. Fig 5(A) shows that the annual citations to the PDB debut paper are higher than the annual counts of mentions of different PDB URLs. Note that since the annual counts were obtained from full-text articles in PubMed Central, we only counted the citations from papers in PubMed Central too for the PDB debut paper here so that the numbers are comparable. Though the annual counts of URL mentions are low, they grow as fast as the citations, which drop in 2013 while the counts of URL mentions continue growing. Fig 5(B) shows that the sum of the annual counts of mentions grows steadily and in 2013 surpasses the citations to the PDB debut paper in that year. The figure also shows the annual counts of the papers that not only cite the PDB debut paper but also mention one of the PDB URLs. Nearly all authors who cited the PDB debut paper did not mention any PDB URL (94%), while authors who chose to directly mention the PDB URLs rarely cite the PDB debut paper (87%). In other words, authors chose to either cite the PDB debut paper or mention URL but rarely do both.

We next consider mentioning of URL as a challenger and investigate whether it disrupts the influence of the PDB debut paper. Here, the citation cascade of the URL mentioning is different from a paper citation cascade only in that its roots are those papers with PDB URL mentions. Then the cascade expands with papers citing these roots and papers citing those citing roots and so on to constitute the citation cascade. We also consider the seven PDB follow-up papers published in the Database Special Issue of NAR between 2002 to 2008 shown in Table 2 collectively as a challenger to compare their disruption impact with the URL mentioning.

**Fig 6:**
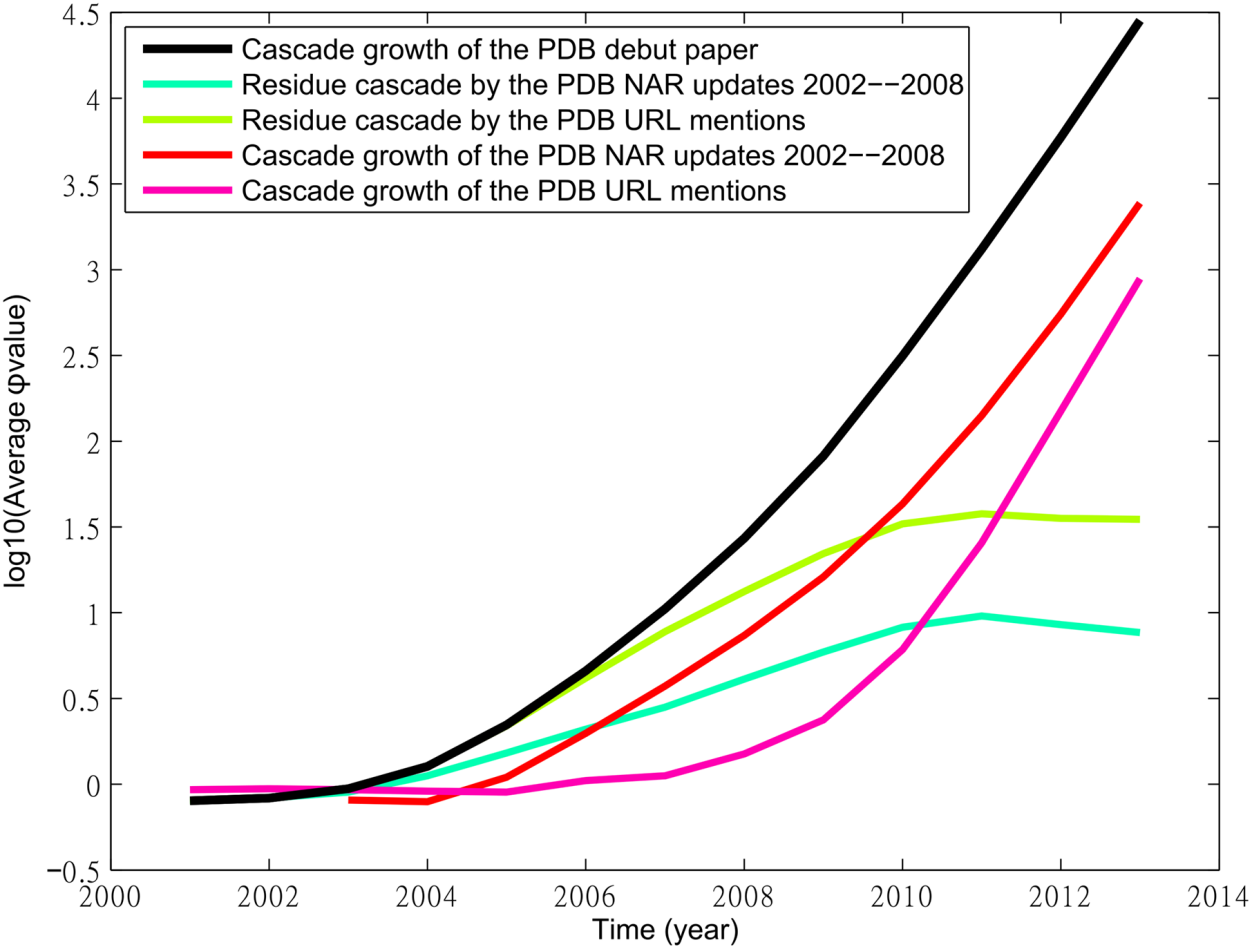
Growth of the cascade of the debut PDB article (black curve), the collection of PDB NAR update articles from 2002 to 2008, the PDB URL mentions articles, and their corresponding residue cascades. Notice the split between the black curve and green curve, indicating the cascade disruption.

Fig 6 plots the growth of the cascades of the PDB debut paper, NAR follow-up papers, and URL mentioning, as well as the growth of the residue cascades by the follow-up NAR papers and URL mentioning. Again, the wider the gap between the curve for the PDB debut paper and the curve of a residue cascade, the higher the disruption of the influence. The figure shows that the gap of the residue cascade of the NAR follow-up papers is also taller than that of the URL mentioning, suggesting that the NAR follow-up papers collectively pose a higher disruption impact to the PDB debut paper than the URL mentioning, though individually, their impact is not apparent. Meanwhile, the growth curve of the NAR follow-up papers rises faster than the curve of the URL mentioning, but the latter is catching up rapidly after 2010.

### Data Usage Statistics

**Fig 7:**
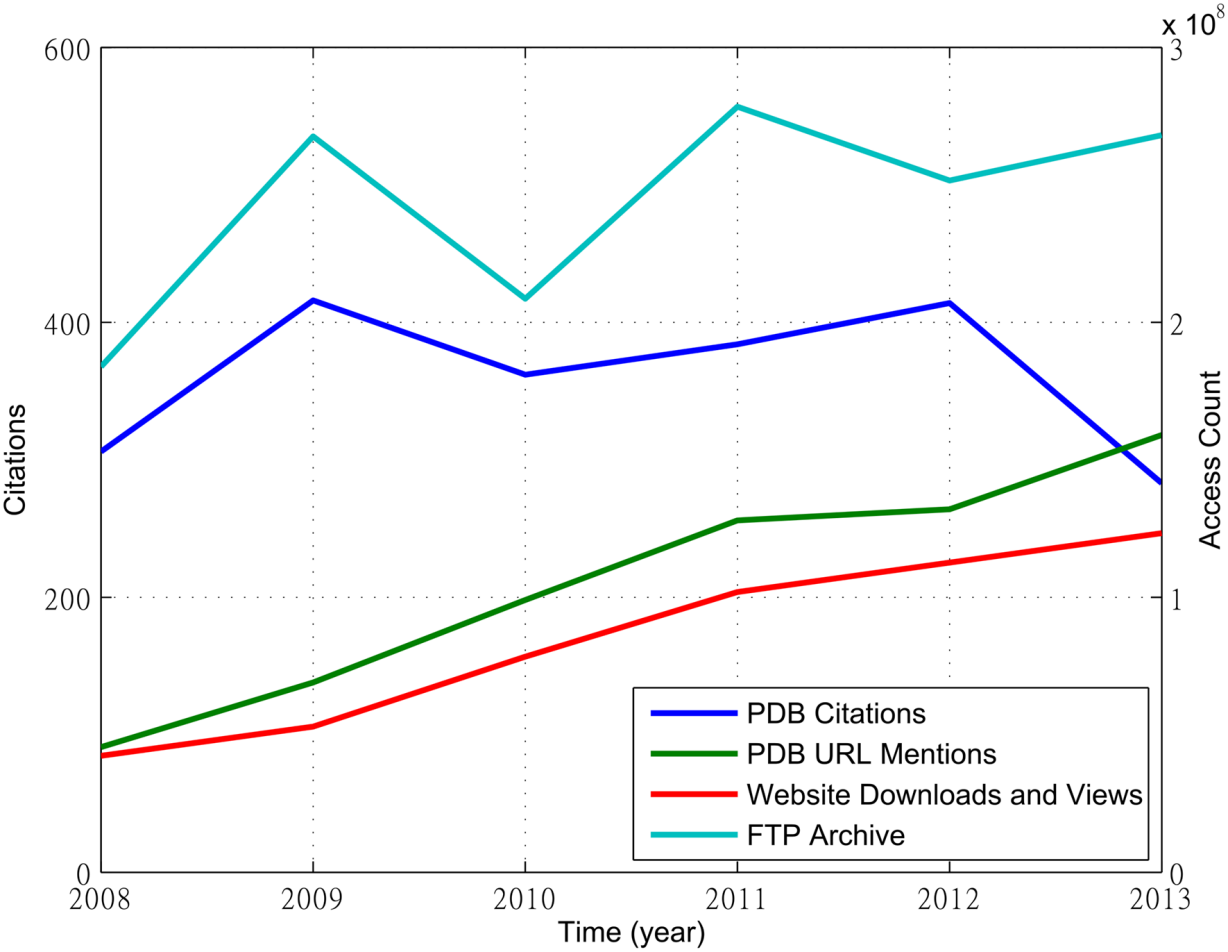
The growth of citations of the PDB debut paper, PDB URL mentions, website downloads and views, and FTP archive access from 2008 to 2013. This analysis only considers citations and mentions available from the PubMedCentral archive.

Various data usage statistics may provide alternatives to citation counts as metrics of impact of a data repository. Yet it is not clear whether these statistics and citation counts are correlated or not. Fig 7 shows that the annual counts of PDB FTP archive access and the citations to the PDB debut paper appear highly correlated before 2013, when the count of citations to the PDB debut paper drops, while the counts of PDB website downloads and views and the counts of the PDB URL mentions appear highly correlated as they grow at a similar rate. Other pairs appear uncorrelated.

**Fig 8:**
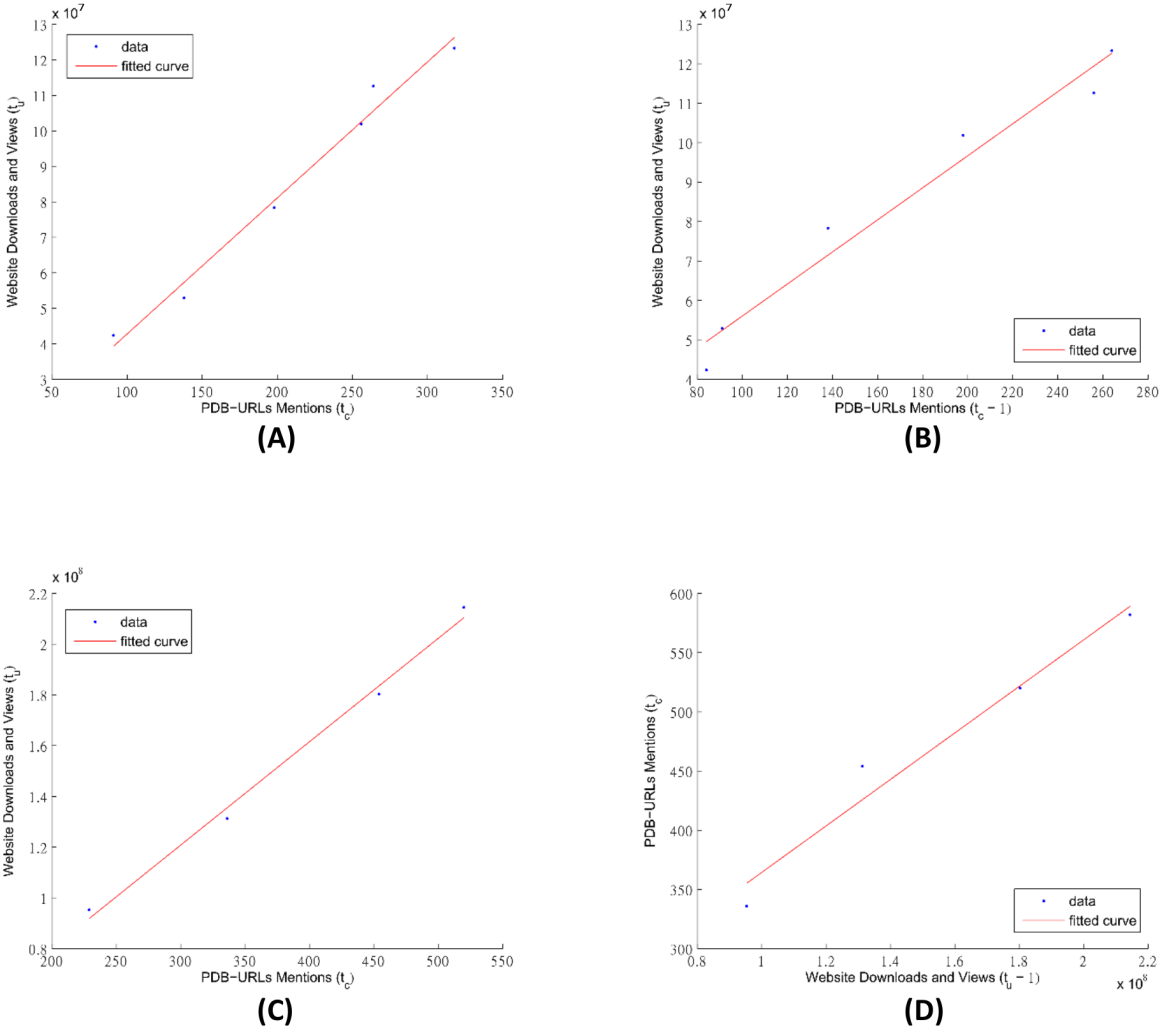
The plots of the fitting of linear models between the PDB URL mentions *c* and the website downloads and views *u*, referred to by their case No.’s in Table 4: (A)Case No. 3, *y* = *u*(*t*), and *x* = *c*(*t*), (B)Case No. 7, *y* = *u*(*t*), and *x* = *c*(*t* − 1), (C)Case No. 11, *y* = *u*(*t*) + *u*(*t* + 1), and *x* = *c*(*t*) + *c*(*t* + 1), (D)Case No. 19, *y* = *c*(*t*) + *c*(*t* + 1), and *x* = *u*(*t* − 1) + *u*(*t*).

We fit linear models to confirm and quantify the observed correlations. Table 4 shows the results of pairing data citations (including both paper citations and URL mentions) and data usage statistics (including both website and FTP access) as either dependent variable or independent variable with different time frames. For example, row No. 19 in the table shows the result of fitting the linear model:

$$\begin{array}{c} c(t)+c(t+1)=w\cdot (u(t-1)+u(t))+\beta , \end{array}$$ (6)

where *c*(*t*) + *c*(*t* + 1) is the sum of the counts of data citations by PDB URL mentions of the current and next year and serves as the dependent variable in the model, *u*(*t* − 1) + *u*(*t*) is the sum of the access counts of the website downloads and views of the previous year and this year and serves as the independent variable to predict the dependent variable, and *w* and *β* are the model parameters that we fit from the data. We quantify the fitness of all results with the *R* ^2^ value. The results show that regardless of the settings the PDB URL mentions and the website downloads and views are highly correlated with *R* ^2^ > 0.9 (in bold fonts). The best fit was found between the two-year sum of the counts of the website downloads and views and the URL mentions (row No. 11). Fig 8 shows the fit of these four cases.

**Table 4:** The correlations between PDB data citations and PDB data usage statistics by linear modeling.

No.	Dependent Variable (*y*)	Time Frame	Independent Variable (*x*)	Time Frame	*R* ^2^
1	Website Downloads and Views	*u*(*t*)	PDB Citations	*c*(*t*)	0.01
2	FTP Archive	*u*(*t*)	PDB Citations	*c*(*t*)	0.13
3	**Website Downloads and Views**	*u*(*t*)	**PDB URL Mentions**	*c*(*t*)	**0.98**
4	FTP Archive	*u*(*t*)	PDB URL Mentions	*c*(*t*)	0.41
5	Website Downloads and Views	*u*(*t*)	PDB Citations	*c*(*t* − 1)	0.67
6	FTP Archive	*u*(*t*)	PDB Citations	*c*(*t* − 1)	0.13
7	**Website Downloads and Views**	*u*(*t*)	**PDB URL Mentions**	*c*(*t* − 1)	**0.97**
8	FTP Archive	*u*(*t*)	PDB URL Mentions	*c*(*t* − 1)	0.30
9	Website Downloads and Views	*u*(*t*) + *u*(*t* + 1)	PDB Citations	*c*(*t*) + *c*(*t* + 1)	0.49
10	FTP Archive	*u*(*t*) + *u*(*t* + 1)	PDB Citations	*c*(*t*) + *c*(*t* + 1)	0.71
11	**Website Downloads and Views**	*u*(*t*) + *u*(*t* + 1)	**PDB URL Mentions**	*c*(*t*) + *c*(*t* + 1)	**0.99**
12	FTP Archive	*u*(*t*) + *u*(*t* + 1)	PDB URL Mentions	*c*(*t*) + *c*(*t* + 1)	0.88
13	PDB Citations	*c*(*t*)	Website Downloads and Views	*u*(*t* − 1)	0.26
14	PDB Citations	*c*(*t*)	FTP Archive	*u*(*t* − 1)	0.08
15	**PDB URL Mentions**	*c*(*t*)	**Website Downloads and Views**	*u*(*t* − 1)	**0.91**
16	PDB URL Mentions	*c*(*t*)	FTP Archive	*u*(*t* − 1)	0.28
17	PDB Citations	*c*(*t*) + *c*(*t* + 1)	Website Downloads and Views	*u*(*t* − 1) + *u*(*t*)	0.26
18	PDB Citations	*c*(*t*) + *c*(*t* + 1)	FTP Archive	*u*(*t* − 1) + *u*(*t*)	0.55
19	**PDB URL Mentions**	*c*(*t*) + *c*(*t* + 1)	**Website Downloads and Views**	*u*(*t* − 1) + *u*(*t*)	**0.96**
20	PDB URL Mentions	*c*(*t*) + *c*(*t* + 1)	FTP Archive	*u*(*t* − 1) + *u*(*t*)	0.89

## Discussions and Conclusions

In this study, we compare data citations to a data repository by citing original and follow-up publications and URL mentioning by applying an approach using disruptions of citation cascades and correlate data citations with data usage statistics for PDB, one of the most widely used biomedical data repositories. Our findings include that

1. Authors still prefer citing the original PDB debut paper to citing follow-up papers.
2. The number of authors citing PDB by URL mentioning is growing rapidly.
3. The impact of PDB URL mentioning, however, is still lower than that of PDB follow-up papers collectively.
4. PDB website access statistics and URL mentions are highly correlated.
5. Correlations between PDB data usage statistics and PDB paper citations are not as high, though PDB FTP access seems to correlate with paper citations in early years.

These trends may be in part the result of the citation policy of the RCSB PDB, which recommends the original PDB debut paper and the URL http://www.rcsb.org as the data resource reference. Since the citation network could be pretty large and could be obtained from different data source, the major technical challenge is to collect a complete set of citation network. Also it can be challenging to integrate the Pubmed citation data with the PMC full-text data for comparing the citing or mention behaviors of PDB users. The analysis of citation trends of other biological data resources with different citation policies will be analyzed in the future to explore this effect and to develop recommendations for data citation practices.

Our analysis methodology is applicable to analyzing citations of Web servers as long as a web server has primary publications that can be used as the root nodes of citation cascades and maintains Web access logs to correlate with citation counts and/or URL mentions.
